# Predicting allergy and postpartum depression from an incomplete compositional microbiome

**DOI:** 10.1186/s12864-025-12390-3

**Published:** 2025-12-06

**Authors:** Andrey Shternshis, Bangzhuo Tong, Alkistis Skalkidou, Carolina Wählby, Dave Zachariah, Luisa W. Hugerth, Prashant Singh

**Affiliations:** 1 Department of Information Technology, Uppsala University, Box 337, Uppsala, 75105 Sweden; 2 Science for Life Laboratory (SciLifeLab), Uppsala University, Uppsala, Sweden; 3 Department of Medical Biochemistry and Microbiology, Uppsala University, Box 582, Uppsala, 75123 Sweden; 4 Department of Women’s and Children’s Health, Uppsala University, Uppsala, 75185 Sweden

**Keywords:** Imputation, Gut microbiome, Compositional data, Log-transformation, Forecasting

## Abstract

Time series of compositional data are a common format for many high-throughput studies of biological molecules, e.g., analyzing the response to a treatment or with the aim of predicting an outcome. However, data from some time points may be missing, which reduces the size of the complete dataset. We propose a method for binary classification that includes imputation for missing values, dimensionality reduction, and logarithmic transformation of compositional data. Imputation approaches entail models that incorporate artificial data alongside true measurements, thereby supplementing the dataset. In the application part, we consider two case studies with longitudinal data and associated target labels, aiming to improve prediction accuracy. We predict infants’ food allergies from their gut microbiome with a balanced accuracy of 0.72. We forecast postpartum depression based on gut microbiome data collected during pregnancy, with a balanced accuracy of 0.62. Features extracted from the microbiome time series, specifically ratios of bacterial abundance, are statistically significant indicators of depression.

## Introduction

Compositional data are a common form of dataset in various fields, including geology [1], demography [2] and chemometrics [3, 4]. In biology, essentially all data derived from sequencing are compositional, being limited to sequencing depth and generally deprived of absolute quantification [5, 6]. Compositional data lie within the probability simplex, meaning that all the elements are positive and sum to 1 (100%). This work focuses on classification problems where the input is a time series of compositional data and the target consists of binary labels (e.g., healthy, diseased). Time series of compositional data essentially arise when data from a study participant are collected periodically, for example, in gene expression profiling [7], exposomics [8], and microbiome studies [9].

The goal of this study is twofold. First, we aim to develop an algorithm for effective binary classification when the input space consists of time points of compositional data. Second, we propose approaches for imputing missing values. Data may be missing at certain time points because of loss of follow-up, failure in sampling or DNA extraction. As a result, it is common in longitudinal studies to have a majority of participants with at least one time point with missing values. Therefore, techniques to impute artificial compositional data at missing time points to enable the use of incomplete data during classification are needed. By imputation, we complement all classes of the data. This results in (1) increasing the size of training data on which classifiers and feature selection algorithms learn and (2) improving diversity of the data by including participants who missed their appointments for any reason. Another application for imputation methods in case of imbalanced data is (3) balancing the dataset by introducing imputed values only for a minor class. Classifiers tend to identify correctly the major class and thus balancing the sizes of two classes is especially important when the amount of cases is less then the controls.

We test proposed methodology on two case studies. Both datasets are gut microbiome data representing the fraction, or relative abundance, of various bacterial species. The classes that we target to predict are food allergies for children and postpartum depression for women. Food protein-induced allergic proctocolitis (FPIAP) is a commonly recognized food allergy in early infancy that is diagnosed in 17% of cases [10]. FPIAP is usually identified on the basis of a positive response to the removal of a food allergen. However, incorrect diagnoses can lead to unnecessary dietary changes for infants [11]. Between 12% and 20% of mothers experience postpartum depression within the first three months after childbirth [12, 13]. O’hara and Swain [14] identified a relationship between postpartum depression and low social support, stressful life events, and psychological disturbances during pregnancy. Identifying such predictors can help detect depression earlier and facilitate timely treatment [15]. In addition to these predictors, we explore the potential of using the gut microbiome as a feature space for forecasting depression.

The paper is organized as follows. First, we present an overview of methods applicable for classification of time points of compositional data. Features (e.g., species) important for classification may be located at different time points, as well as present changes in values over time. Second, we impute missing compositional data comparing various imputation methods. These methods are used to enlarge the input space for training classification models and to balance the dataset. For the datasets considered, imputation improves classification sensitivity and balanced accuracy. We achieve the prediction accuracy of 0.72 for FPIAP using data from the first six months of life. The sensitivity is 0.66 and the specificity is 0.78. Finally, we demonstrate that the gut microbiome of pregnant women contains statistically significant features for classifying postpartum depression. By balancing the data, we achieve the balanced accuracy of 0.62 with a corresponding sensitivity of 0.39 for forecasting postpartum depression at six weeks after childbirth based on data collected during pregnancy.

The next section reviews relevant papers on metrics, models, and applications. Methods section presents the methods for data transformation, imputation, and classification. Structure of the data and datasets section introduces the datasets. Results section presents the results. Discussion section concludes the paper.

## Background

Brodersen et al. [16] introduced balanced accuracy, which averages the accuracies for each target group. Given a confusion matrix for binary classification with the number of positives (*P*), true positives ($T_P$), number of negatives (*N*), and true negatives ($T_N$), the balanced accuracy is defined as follows:

$$\begin{aligned} acc = \frac{T_P}{2P} + \frac{T_N}{2N}, \end{aligned}$$

where $T_P/P$ is referred to as *sensitivity*, and $T_N/N$ is referred to as *specificity*. Random or constant decisions result in a balanced accuracy of $acc = 0.5$. In binary classification, balanced accuracy is equivalent to the area under the receiver operating characteristic curve [17].

Xia et al. and Okazaki, Kawano [18, 19] proposed a regression model for classification using logarithmic ratios of compositional data and a lasso regularizer for feature extraction [20]. Logarithmic ratios allow the transformation of compositional data into real space [21, 22]. Research on 24 datasets and a subset of compositional data transformations [23] concluded that transformations have a limited effect on classification accuracy.

Martin et al. [11] analyzed the taxonomic differences in the gut microbiome between infants with food protein-induced allergic proctocolitis (FPIAP) and control cases (no FPIAP). The authors applied a random forest classifier to evaluate feature importance [24] and determine prediction accuracy. When distinguishing control samples from three stages of FPIAP development (resolved, symptomatic, and presymptomatic), the balanced accuracy reached 0.53. In the case of the four groups, the balanced accuracy ranges from 0 to 1, with a value of 0.25 corresponding to a random guess. In a simpler scenario, when only samples from infants aged six months were used within the control or resolved groups, the balanced accuracy improved to 0.66, with a sensitivity of 0.76 and a specificity of 0.56.

Andersson et al. [12] used pregnancy- and childbirth-related variables along with psychometric questionnaires to predict postpartum depression. The authors achieved a balanced accuracy of 73% and a sensitivity of 72%. According to their findings, the most important variables for prediction include depression and anxiety during pregnancy as well as a history of depression. Postpartum depression primarily involves symptoms of anxiety and depression, which are closely correlated with changes in the gut microbiome [25]. Moreover, several studies have reported changes in gut microbiome abundance in individuals with postpartum depression [26, 27]. Tortajada et al. [28] achieved a balanced accuracy of 83% and a sensitivity of 84% using background data, emotional alterations, and depressive symptoms. Zhang et al. [29] obtained a balanced accuracy of 85% and a sensitivity of 82% using data extracted from electronic health records after childbirth.

## Methods

### Structure of the data and datasets

We analyze the data in the following form: each participant’s data consists of *T* time points (tp) of compositional data. Measurements, represented as probability vectors (relative abundance summing up to 100%), are collected multiple times for each participant. Labels are associated either with individual time points or with the entire time series. Compositional data may be missing at certain time points. A sketch of the data is shown in Table 1. In the provided example, there are *n* data points, and we consider the case where $T=3$. However, this is not a limitation for the classification and imputation approaches discussed in the next section.

**Table 1:** Sketch of the dataset

datum id	tp	fractions			label
		fraction of element 1		fraction of element d	
1	1	$p_{1,1,1}$	...	$p_{1,1,d}$	−1
	2	$p_{1,2,1}$		$p_{1,2,d}$	1
	3	$p_{1,3,1}$		$p_{1,3,d}$	1
$$\cdots$$					
n	1	missing			
	2	missing			
	3	$p_{n,3,1}$	...	$p_{n,3,d}$	−1

$\sum _{k=1}^{d}p_{i,j,k}=1$, where$0\le p < 1$, tp denotes the time point and id denotes the number of a participant/case

The analysis is conducted on two datasets described below.

Dataset 1: The BASIC study [30] at Uppsala University Hospital collected data from pregnant women between 2009 and 2018. The participants were asked to complete the Edinburgh Postnatal Depression Scale (EPDS) questionnaire [31]. Starting in 2016, gut microbiome data were included as part of the input space. The compositional data represent relative species abundance, with a dimension of $d = 713$ species in the gut microbiome. Data were collected at three time points: during the 20th and 30th weeks of pregnancy and at six weeks postpartum. The outcome for each time point is the presence of depression, as defined by the EPDS. The label to forecast is at the last time point ($t = T$). For binarization, participants are considered healthy ($y = -1$) if their EPDS score is less than 12 and depressed ($y = 1$) otherwise [32]. These data have been previously described in [33].

For complete data with no missing values, the dataset includes 82 data points with $y = -1$ and only 15 data points with $y = 1$, indicating a relatively small and imbalanced dataset. For data points where the last time point is missing, there are 16 positive and 108 negative labels. For a subset where the first or second time point is missing, there are 33 positive and 190 negative labels. A summary of the sizes is given in Table 2.

**Table 2:** Amount of data points available for Dataset 1

labels/time points	1,2,3	1,2	1,3	2,3	1	2	3
$y = -1$	82	108	42	30	57	61	13
$y = 1$	15	16	4	4	12	13	1

Numbers indicate how many data points are available with the label (rows) and not missing time points (in columns)

Dataset 2: FPIAP. The second study examined the relationship between the gut microbiome and food allergies [11]. It involves 80 healthy infants ($y = -1$) and 82 infants diagnosed with food protein-induced allergic proctocolitis (FPIAP) during their first year of life ($y = 1$). The microbiome was sampled at multiple time points, specifically at the ages of 1, 2, 4, 6, 9, and 12 months. Table 3 summarizes the number of data points available at several time points. The amount of available data decreases as children approach one year of age. We omit three columns from the dataset that are associated with unassigned bacteria, reducing the dimensionality of the compositional data to $d = 342$.

**Table 3:** Amount of data points available for Dataset 2

labels/time points	1,2,4	2,4,6	4,6,9	1,2	1,4	2,4	2,6	4,6	4,9	6,9
$y = -1$	31	30	20	46	36	42	41	37	32	30
$y = 1$	30	20	17	47	36	37	32	25	28	30

Numbers indicate how many data points are available with the label (rows) and not missing time points (months in columns)

Having discussed the format of the data, we now provide an overview of the methods applied for classifying data points by their target labels. This section begins with ways for dimensionality reduction. Next, we discuss data transformations that enable us to work in real space instead of the original compositional data format. We then introduce imputation techniques to handle incomplete time points, thereby enlarging the training set. Finally, we explore several classification approaches. When specific time points are used for classification, we assume that the data are either complete or that missing time points have been imputed via the methods outlined in Imputation section.

The data are divided into training, validation, and test sets. We apply nested cross-validation procedure that returns stratified folds. That is, the imbalance ratio of labels remains consistent between groups across all sets. When test set is separated from the data, the rest is divided into training and validation sets. We randomly divide data into 5 equal-size test sets. The remaining data are divided into 5 cross-validation sets. That is, the proportions of test, training, and validation sets are 0.2, 0.64, and 0.16, respectively. For instance, this results in 3 and 2 data points with positive labels for the test and validation sets in Dataset 1, respectively. We use validation sets to identify the optimal number of features selected as discussed in Feature selection section. The features are constructed as shown in Data transformation section. The accuracy of prediction is calculated on test sets.

### Feature selection

Feature selection is a fundamental approach of dimensionality reduction allowing us to select the most relevant features and potentially improves prediction performance and data interpretability. The goals of feature selection are (1) to avoid overfitting of models, (2) to provide faster realization of methods, (3) to enhance comprehensibility of results and, (4) to remove redundant features [34, 35].

The features are ranked by the F-statistics on F-test in analysis of variance (ANOVA) [36]. It measures the degree of separability of two groups features divided by labels. The larger variability between groups relative to variability within groups, the larger the F-statistics. Features with the highest F-statistics are chosen.

The alternative methods for comparison analysis are chosen to be Lasso regularization [20], mutual information (MI) [37], and principal component analysis (PCA) [38]. The optimal number of features is defined by the maximum mean/median of the classification accuracy on validation sets. We also test another approach for dimensionality reduction by ranging features by their detection frequency. We assume that a common outcome cannot depend directly on an uncommon exposure. Species with a sparsity greater than a predetermined threshold are combined into a group called “other species.” Sparsity refers to the fraction of zero values across the training set calculated for each species. Depending on the threshold value, we can significantly reduce the dimensionality of the problem, although potentially important information may be lost by merging sparse species.

### Data transformation

Each data point is two-dimensional, containing compositional data at several time points. By logarithm transformations, we avoid compositional restriction and transfer the data into real space. Taking differences between logarithms, we consider relations between species and time points. We consider four ways of transforming the data.

**Fig. 1:**
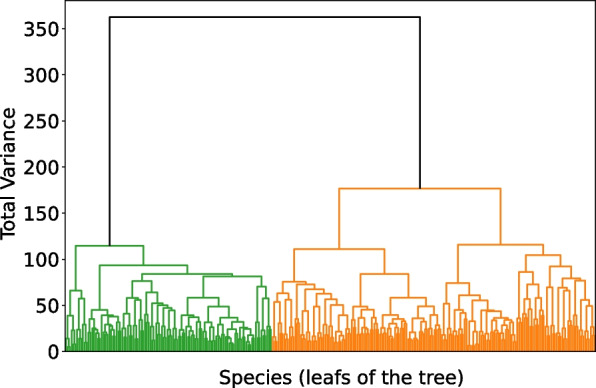
Example of hierarchical tree of a training set of merged species

- *Compositional*. Each element $x_{i,t}$ is considered an input feature, where $1 \le i \le d$ represents the number of species and $1 \le t \le T$ represents a time point.
- *Centered* log ratios (CLR, [39]). Each element at a time point is divided by its geometric mean. The logarithm of these ratios is called CLR:
  $$\begin{aligned} CLR_{i,t} = \log {\frac{x_{i,t}}{g_t}}, \end{aligned}$$
  where $g_t = \left( \prod _{i=1}^d{x_{i,t}}\right) ^{1/d}$. This transformation shifts the data from a compositional structure to a real space.
- *All* log ratios (ALR). We flatten data points to compositional data by concatenating time points and dividing by *T*.
  $$\begin{aligned} X= & [X_1, X_2, \dots , X_{dT}],\\= & [x_{1:d, 1}, x_{1:d, 2},\dots , x_{1:d, T}]/T, \\ ALR_{i,j}= & \log {\frac{X_i}{X_j}}, j>i. \end{aligned}$$
  In this way, we increase the feature space dimension from $D = dT$ to $\frac{D(D-1)}{2}$. With this transformation, we assume that the target labels are influenced not only by the fractions at individual time points, but by how bacteria change over time. A dummy constant element may be introduced to add logarithms of compositional elements to the input space together with all ratios.
- *Pivot* log ratios (PLR). The log ratios between clusters of elements are used here. The number of all possible clusters is large; thus, we limit it by pivot features, as suggested in [40]. The clustering metric is the minimum variance within a cluster. It is known as the Ward method [41]. The PLR is the ratio of the geometric means of the left and right subtrees for each node. We set the first feature to be the geometric mean, *g*(*X*). As shown in Fig. 1, the second feature is the ratio of the geometric means of the left (green) and right (orange) trees. All other subtrees are defined iteratively. With the PLR transformation, we do not increase dimensionality and retain the same amount of information as in the original dataset. Furthermore, it is possible to identify the specific clusters of bacteria used to calculate each feature. Trees are constructed using only seen data, i.e. training set only for determining the optimal number of features or training and validation sets together to test the classification accuracy.

For the logarithmic transformations, all the elements must be positive. We identify the minimum value in the training set and replace all zero values with half of this minimum value, ensuring that the sum remains equal to 1. We assume that all species are present in all samples, at least in very low abundance since all data is derived from the same population, and that metagenomic sequencing data is seldom saturated. All transformations of training data are not affected by validation and testing sets. Alternatives for a data transformation that allows 0 values include [42, 43].

### Classification

We select random forest [24] as a commonly used classifier [44, 45]. We compare it with the support vector classifier (SVC, [46, 47]) and adaptive boosting (AdaBoost, [48]), which are known state-of-art methods for binary classification. Both random forest and AdaBoost are based on decision trees. A decision tree partitions data along different routes capturing distinct pathways that converge to the same health condition. Random Forests build many such trees and combine their results, so they can recognize multiple combinations of values leading to the same biological outcome. AdaBoost adjusts sample weights based on the classification outcomes of the decision trees. Approaches that explicitly control classification errors conditioned on a class are also considered, as discussed in [49].

In results section, we present *p*-values for one-sample t-test [50] evaluating whether the mean balanced accuracy achieved by a classification pipeline significantly exceeds 0.5. For multiple imputations described in the next section, another test is applied and correction for variance is done. We also report the *p*-values from the Welch’s t-test [51] for equal means of the method with the highest balanced accuracy and other methods in the same table.

### Imputation

Imputation refers to the process of replacing missing data with artificially generated values. We employ five imputation approaches that use available time points to restore the missing points. A common step for all approaches is to transform the compositional data at each time point into a CLR. This transformation has a well-known inverse, called Softmax [21]. The approaches differ in how they select the best-fit simulated predictions for imputation. In other words, we present various models with different objective functions for optimization listed below.

- A matrix *A* and a vector *b* are chosen to minimize the least squares error between $AC_n + b$ and $C_m$, where the output $C_m$ represents a missing time point and the input $C_n$ represents time points that are not missing. The training set is used to find the optimal parameters. We consider linear regression as a baseline for comparison with more sophisticated models. A simpler model could be to substitute equal values instead of all missing fractions.
- Support Vector Regression (SVR, [52]). SVR is a nonlinear regression technique that aims to find a function that best fits the data by minimizing the prediction error within a specified margin. The optimization does not depend on the input dimensionality, allowing SVR to handle complex nonlinear relationships in the data.
- Gaussian process regression (GPR, [53]). The approach provides a probabilistic approach that considers data as a realization of a stochastic process. The values imputed have the maximum likelihood. GPR treats models as distributions over functions. Predictions are made via the mean of the Gaussian process.
- Conditional Variational Autoencoder (CVAE, [54]). Another probabilistic approach allows the restoration of missing values from white noise. During training, noise is encoded from all given time points. The prediction is decoded from inputs and a noise term.
- Conditional generative adversarial network (cGAN, [55]). Another way to learn a generative neural network. In this paradigm, the goal is to create predictions indistinguishable from real data by a discriminator. Similar to the previous approach, predictions are generated from a noise term and available data allowing for nonuniqueness of the imputed values.

**Fig. 2:**
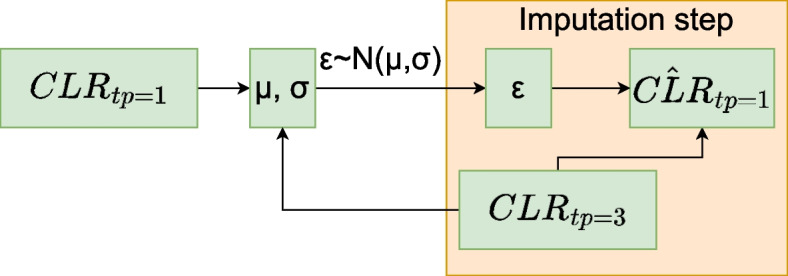
Example of the CVAE architecture where tp 1 is reconstructed from tp 3 and random noise $\epsilon$. During imputation, $\epsilon$ is sampled from Gaussian *N*(0, 1)

**Fig. 3:**
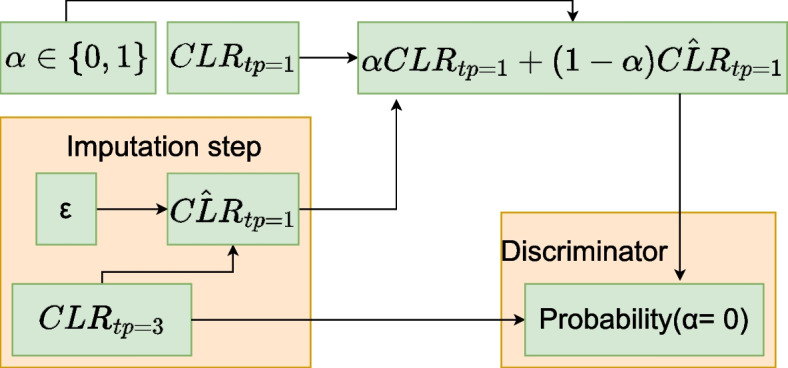
Example of cGAN architecture where tp 1 is reconstructed from tp 3 and random noise $\epsilon$, is sampled from Gaussian *N*(0, 1)

See Figs. 2 and 3 for the architectures of Neural Networks. See Hyperparameters section for implementation details.

The last three approaches have a random nature in parameter initialization and random noise generation. Thus, we run 5 imputation sets for each testing set. Multiple rounds of imputation highlight the uncertainty around the imputed values [56]. When several rounds of imputation are done, we make a correction in variance estimation according to [57]. In this case, we apply Wald test [58] to calculate *p*-values for hypothesis testing that the value of balanced accuracy is higher than 0.5.

We acknowledge that incomplete datasets with imputed values may have distributions that differ from those of complete datasets. To address this, we augment the input space with binary class variables indicating whether the data at each time point were imputed.

### Hyperparameters

The remainder of section is dedicated to the hyperparameters chosen for the experiments.

- The threshold for sparsity is 0.5 for Dataset 1 and 0.9 for Dataset 2. That is, we combine bacteria into one group for dimensionality reduction if their sparsity is greater than one half for the dataset regarding depression.
- Since the optimal number of features defined by cross-validation rarely exceeds 10, we set the maximum amount of features to 20.
- For the lasso regularizer, we set a random state of 10, an $L_1$-norm weight of 10, and increase the maximum number of iterations to $10^4$ to ensure convergence of the total loss.
- The default options for the random forest classifier are used from the sklearn.ensemble library in Python [59].
- Standard scaling is applied before support vector regression: linear transformation is done so that the mean of features in training set is 0 and the standard deviation is 1. The default parameters of sklearn.svm.SVR use a radial basis function (RBF) kernel, with a margin of 0.1 within which no penalty is applied.
- sklearn.gaussian_process.GaussianProcessRegressor is used for GPR. RBF kernel is used. The number of restarts of the optimizer is set to 5. The random state is iterated from 0 to 4. All other parameters are default.
- To limit the number of parameters to train due to the small dataset size, we consider neural networks with one hidden layer in each of the components of the neural networks. The size of hidden layer is equal to the output’s dimension. The size of the latent space, which is used to generate random noise, is set to 1. We use a batch size of 1, with a maximum of 1000 epochs. The stopping criterion is based on an increase in validation error, meaning that the “patience” parameter is set to 0. To train the neural networks, the default settings of the Adam optimizer [60] from the torch.optim library [61] are used. Sigmoid activation function is used for the discriminator of cGAN, otherwise hyperbolic tangent is applied. We use 5 random seeds with values from 0 to 4.

## Results

We start by testing log-transformation on a complete part of the dataset regarding postpartum depression. Log transformations are used for converting input compositional data from the (0, 1) range into the real number space. We then proceed with forecasting the depression by analyzing a reduced feature space and applying imputation approaches in Forecasting depression section. The results of log-transformation and imputation techniques for the allergy dataset are given in Prediction of food allergies section.

### Classification of depression

First, we present the results of a classification problem using data points from Dataset 1 that have no missing time points, including the last one after childbirth, which is available for analysis. We apply data transformation through log ratios, as described in Data transformation section. We also examine whether dimensionality reduction by merging bacteria, as detailed in Feature selection section, improves performance. Given that the data are imbalanced, we investigate whether assigning different weights during classification enhances accuracy. The optimal number of features, *n*, is selected through cross-validation. The balanced accuracies from the validation sets are averaged using either the mean or median. The median is more robust to random perturbations, often resulting in a smaller optimal number. The results are averaged over 5 runs of the classifier with different random seeds. The mean results from the 5 testing sets, including balanced accuracy (acc), sensitivity (sens., correctly identified depression), and specificity (spec., correctly identified no depression), are presented in Table 4. We report the interval defined by ±1.96 square roots of variance from the mean, which corresponds to a 95% confidence interval under the assumption of normality.

We conclude from the resulting table that the highest accuracy is achieved with dimensionality reduction by merging species. Adjusting weights according to class size does not appear to be beneficial. These choices for merging (on) and balancing (off) are fixed for the remainder of the study. Other user options, such as classifier selection and feature selection, are detailed in Appendix A.

**Table 4:** Accuracy for different types of log ratios for Dataset 1

log ratio	merging	balance	metric	acc ± 1.96std	*p*-value	sens.	spec.	n
Compositional	on	on	mean	0.55, (0.38, 0.71)	0.01, 3e-5	0.13	0.96	5,6,3,2,2
Centered	on	off	median	0.55, (0.39, 0.71)	0.01, 3e-5	0.16	0.93	9,2,6,1,14
		on	median	0.53, (0.38, 0.68)	0.03,4e-6	0.13	0.93	8,2,5,1,8
All	off	off	mean	0.55, (0.28, 0.82)	0.04, 9e-4	0.13	0.97	3,4,5,1,5
	on	off	mean	0.53, (0.42, 0.63)	0.001, 1e-6	0.13	0.92	7,4,1,4,13
			median	0.53, (0.4, 0.67)	0.01, 4e-6	0.09	**0.98**	6,3,11,2,3
Pivot	on	off	mean	0.67, (0.38, 0.96)	6e-6, 0.64	0.39	0.94	4,3,2,5,1
			median	**0.68**, (0.45, 0.92)	4e-8,-	**0.4**	0.97	4,1,1,1,2
		on	median	0.64, (0.32, 0.94)	2e-4,0.24	0.33	0.94	2,1,1,1,1

Two *p*-values correspond to one-sample and Welch’ tests. Only results with *p*-value on one-sample t-test less than 0.05 are shown. If Merging is on, sparse bacteria are merged. If balance is achieved, class weights are inversely proportional to class frequencies. The best results in each column are highlighted in bold. *n* is the optimal number of features used

The pivot log ratio, which considers differences between clusters of species, outperforms other transformations under analysis. Pivot log ratios allow us to look at the data in terms of differences in bacterial abundance as well as changes over time while maintaining the original dimensionality. Hierarchical trees for PLR are constructed omitting testing sets to avoid data leakage. As shown in Table 4, the optimal number of features, *n*, does not exceed 5. It appears that all features are ratios between two bacteria. Four out of the five test sets show a balanced accuracy, averaged over five runs of classification, greater than 0.5. For these four runs, we present the features used to classify depression with corresponding number of appearance in Table 5. The relationship between these features and the labels is clearly identified in the case of pivot log ratios but not with all log ratios because of the dimensionality of the feature space introduced by the log transformations. We present a feature list when mean is chosen as the metric, which provides a more comprehensive representation. Most of pairs is used for classification only once, but Veillonella parvula and Haemophilus parainfluenzae appears in all cases. Previous studies have shown that Veillonella parvula is positively correlated with major depressive disorder [62] and bipolar disorder [63], whereas low levels of Haemophilus parainfluenzae were associated with psychotherapeutic responses in [64].

**Table 5:** List of bacteria used in the classification of depression

Bacteria 1	Bacteria 2	# appearances (out of 4)	identified by median?
1 Coprococcus comes	1 Dorea longicatena	1	Yes
1 Roseburia faecis	2 Roseburia faecis	1	
1 Eubacterium sp CAG 38	2 Eubacterium sp CAG 38	1	
2 Veillonella parvula	2 Haemophilus parainfluenzae	4	Yes
2 Akkermansia muciniphila	3 Akkermansia muciniphila	1	
3 Ruthenibacterium lactatiformans	3 Dorea formicigenerans	2	Yes
3 Eggerthella lenta	3 Gordonibacter pamelaeae	2	Yes
3 Roseburia intestinalis	3 Eubacterium sp. CAG 38	1	
3 Coprococcus comes	3 Dorea longicatena	1	

Number before each bacteria name is a time point. Number of appearances refers to the number of testing sets out of 4 where the pair of bacteria appears in a feature space. Choosing the optimal number of features by the median reduces the number of features to four, as marked in the last column

Some features from Table 5 represent changes in the abundance of the same bacteria over time. The same pair of bacteria also appears to be important at both time points 1 and 3. Only half of the bacterial species are detected during the last time point (postpartum).

Using only the four features selected by the median in Table 5, we obtain a sensitivity of 0.33 with a specificity of 0.993. When only the first two features, which can be obtained during pregnancy, are used, the specificity improves to 0.995.

Further results on forecasting postpartum depression based on only features obtained during pregnancy follow.

### Forecasting depression

Considering only the first two time points for depression forecasting allows us to include into the analysis data points with missing data at time point 3, i.e. women who missed the last visit. However, this adjunction decreases the balanced accuracy to 0.55, with a sensitivity of 0.17. The result is still statistically significant, with a *p*-value of $4e-4$. That is, the forecasting works better under the assumption that a woman under consideration does not miss the last visit after a childbirth.

Taking the ratio between the abundances of Veillonella parvula and Haemophilus parainfluenzae at two time points during pregnancy as two features, we increased the balanced accuracy to 0.61, with a sensitivity of 0.25. When we augment the training dataset with imputed values, we do not observe an improvement in classification accuracy. The results for various imputation methods are summarized in Table 6.

**Table 6:** Accuracy for different types of imputation for Dataset 1

imputation	tp imputed	acc ± 1.96std	*p*-value	sens.	spec.	mean n
linear regression	1	0.6, (0.46, 0.74)	1e-7, 0.89	0.26	0.95	2.4
SVR	2	0.58, (0.39, 0.77)	3e-4,0.21	0.23	0.93	2
	1	0.58, (0.45, 0.7)	2e-06,0.06	0.19	**0.96**	2.6
GPR	2	**0.61**, (0.4, 0.82)	0.004,0.63	**0.27**	0.95	2.4
	1	**0.61**, (0.45, 0.76)	0.003,-	**0.27**	0.94	2.2
	1,2	0.6, (0.42,0.78)	0.002,0.7	0.25	0.95	3.2
CVAE	2	0.6, (0.41, 0.79)	0.003, 0.65	0.24	**0.96**	2.6
	1	0.59, (0.42, 0.76)	0.001,0.24	0.24	0.95	2.6
	1,2	0.58, (0.39, 0.77)	0.006,0.04	0.21	0.95	2.48
cGAN	2	0.6, (0.42, 0.79)	0.002, 0.84	0.25	**0.96**	2.36
	1	0.6, (0.45, 0.76)	3e-4, 0.83	0.25	0.95	2.48
	1,2	0.59, (0.38, 0.8)	0.001, 0.13	0.23	0.94	2.28

Two *p*-values correspond to one-sample and Welch’ tests. Only results with *p*-value on one-sample test less than 0.05 are shown. Imputing tp 1 and tp 2 from each other is considered. Imputing tp 2 by linear regression gives 0 values of bacterial abundance. The best results in each column are highlighted in bold. *n* is the optimal number of features selected by median

Imputation with the goal of balancing the dataset increases accuracy metrics. When incomplete time points with only positive labels are are imputed by GRP, the balanced accuracy increases to 0.62, with a sensitivity of 0.39. Empirical 95% Confidence interval is (0.52, 0.75). The downside of this data balancing is that we only provide extended data for cases, while leaving controls limited to only the complete sets. However, we fix the testing sets for any type of imputations to make the evaluation fair and thus there is no presence of artificial values in testing sets.

We supplement the analysis on forecasting depression in Table 7. We test if depression can be predicted from microbiome diversity, data missingness, and PPD labels during pregnancy. We find that missing data was not a significant predictor of depression. By using two binary variables denoting the presence of missing values at the time points during pregnancy, we achieve a balanced accuracy of 0.5. In contrast, using depression states during pregnancy together with missingness results in a balanced accuracy of 0.59, with a sensitivity of 0.24. We also calculate Shannon entropy [65] representing the diversity of data when it is available. It gives the mean accuracy of 0.55. We outperform these results by using the gut microbiome.

**Table 7:** Accuracy for different inputs

inputs	acc ± 1.96std	*p*-value	sens.	spec.	mean n
Missingness	0.5, (0.5, 0.5)	1, 0.0	0	1	1
Missingness+PPD	0.59, (0.54, 0.64)	7e-16, 0.0	0.24	0.95	3
Shannon index	0.55, (0.4, 0.7)	0.002, 0.0	0.23	0.87	1

Two *p*-values correspond to one-sample and Welch’ tests. Welch’ tests are done for accuracy results from inputs (first column) and the results obtained by balancing the data through imputation (mean accuracy of 0.62.)

### Prediction of food allergies

By presenting the second dataset, we aim to demonstrate the effectiveness of imputation approaches for prediction tasks and provide a more comprehensive comparison of the different methods. We fix the number of time points and consider three consecutive time points: 2, 4, and 6 months. By merging bacteria at these time points, we achieve a balanced accuracy of 0.58. The impact of different data transformations on performance is less pronounced than the effect of choosing between the mean and the median according to Table 8. Applying a log-ratio transformation to the input space increases the balanced accuracy to 0.66, with a sensitivity of 0.52.

**Table 8:** Accuracy for different types of log ratios for Dataset 2

log ratio	metric	acc ± 1.96std	*p*-value	sens.	spec.	n
compositional	mean	0.59, (0.45, 0.72)	1e-06, 9e-4	0.36	**0.81**	2 14 1 18 14
	median	0.64, (0.47, 0.7)	2e-8, 0.11	0.5	0.77	1 1 1 9 2
All	mean	**0.66**, (0.52, 0.79)	2e-11, -	**0.52**	0.79	16,13,6,14,7
	median	0.59 (0.36, 0.82)	4e-4, 9e-4	0.39	0.79	5 6 6 6 2
CLR	mean	0.55, (0,35 0.74)	0.01, 5e-5	0.32	0.77	2 18 6 20 2
	median	0.59, (0,37 0.82)	3e-4, 0.59	0.44	0.75	7 10 4 10 2

Two *p*-values correspond to one-sample and Welch’ tests. Only results with *p*-value on one-sample t-test less than 0.05 are shown. The best results in each column are highlighted in bold. *n* is the optimal number of features used

According to the study by [11], compositional data are informative with respect to food allergies. When the training set of the compositional data is augmented by data with one-third of the time points imputed by cGAN, the balanced accuracy improves to 0.72, with a sensitivity of 0.66. For a detailed comparison of the imputation approaches, see Table 9. By increasing the size of the training set through the inclusion of incomplete data points, we improve the prediction accuracy. Additionally, we compare imputation methods by masking and then imputing time points in validation sets. The means of Aitchison distance, $D_A$, between the imputed and true time points are computed and reported in Table 8. Aitchison distance is the Euclidean distance calculated between two CLR transformations of compositional data [66]. While the maximum prediction accuracy is achieved by cGAN, the minimum of Aitchison distance is obtained by CVAE.

**Table 9:** Accuracy for different types of imputation for Dataset 2

imputation	data points imputed	acc±1.96std	*p*-value	sens.	spec.	$D_A$	mean n
SVR	all	0.61, (0.38, 0.84)	5e-5,3e-4	0.59	0.63	21.4	8
	with 1 tp missing	0.56, (0.33, 0.79)	0.01, 5e-7	0.52	0.59	21.6	6.8
GPR	all	0.64, (0.38, 0.9)	8e-3, 5e-7	0.55	0.73	21.7	8.8
	with 1 tp missing	0.66, (0.51,0.82)	2e-6,3e-5	0.65	0.68	21.6	8
CVAE	with 1 tp missing	0.66, (0.41,0,9)	3e-3, 1e-4	0.64	0.68	**16.6**	5.68
cGAN	all	0.66, (0.41, 0.92)	3e-3,5e-4	0.58	0.75	23.5	8.32
	with 1 tp missing	**0.72**, (0.49, 0.95)	4e-4,-	**0.66**	**0.78**	22.9	7.24

Two *p*-values correspond to one-sample and Welch’ tests. Only results with *p*-value on one-sample test less than 0.05 are shown. The best results in each column are highlighted in bold. For “all” data points, one or two time points can be missing. In “with 1 tp missing”, two other time points are available. *n* is the optimal number of features selected by mean

There are 24 sets of predictors providing balanced accuracy higher than 0.5 across 5 testing sets and 5 seeds for imputation by cGAN. Bacteria names with corresponding time point appearing in the most of such sets are presented in Table 10. The abundance of Lactobacillus is an important feature for classification at all three time points. All bacteria listed in Table 10 were also identified in the research [11].

**Table 10:** List of bacteria frequently used in the classification of food allergy

Time point	Family of bacteria	Bacteria	# appearances (out of 24)
1	Lactobacillaceae	Lactobacillus	24
2	Lactobacillaceae	Lactobacillus	24
3	Lachnospiraceae	Dorea	21
3	Lactobacillaceae	Lactobacillus	20
3	Lachnospiraceae	Blautia	17
3	Veillonellaceae	Veillonella	16

Features are given in the descending order of number of appearances in subsets for predicting food allergy (last column). Each feature is represented by a time point, bacteria name and its family

**Fig. 4:**
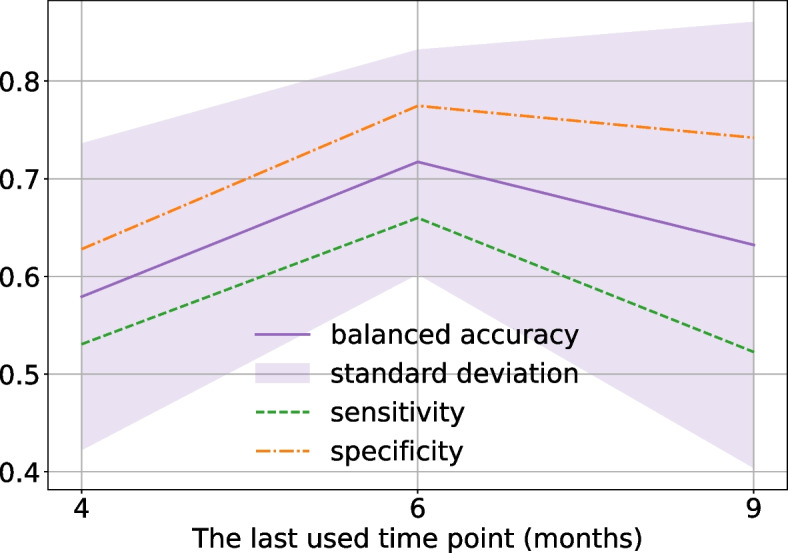
Balanced accuracy on different time points for Dataset 2. The x-axis represents the last time point considered in months of life. The y-axis represents metrics such as balanced accuracy, sensitivity and specificity

To validate the choice of time points, we repeat the imputation and prediction for two alternative sets of months where the microbiome samples are taken: [1, 2, 4] and [4, 6, 9]. The results shown in Fig. 4 demonstrate that the highest balanced accuracy and sensitivity are achieved with the time points at 2, 4, and 6 months. This outcome can be explained by a stronger correlation between the abundance of certain bacteria and food allergies, as well as the lack of complete data for months [4, 6, 9] for training.

## Discussion

### Methodology

We have presented an approach for binary classification when several time points of compositional data are given. We construct the following algorithm for the prediction task when the input is microbiome data. We (i) reduce the dimensionality and sparsity of the data, (ii) impute missing data and merge it with the training set, (iii) transform the data via log ratios, (iv) select the features via analysis of variance on a training set [36], and (v) use the features as the inputs of a random forest classifier [24]. Species with high sparsity are clustered, based on the assumption that a common outcome is unlikely to be driven by a rare exposure. Logarithmic transformations remove compositional limitations from the data that create negative correlations between bacterial species [67]. Pivot log-ratios take into consideration the real correlations in the dataset and use them to find the minimal biological unit used in the transformation. The optimal number of features is chosen via cross-validation. The results are averaged on the testing sets, and imputation inference and initialization parameters are used.

The importance of imputation when some time points are missing can be considered from different perspectives: (i) we increase the size of the training set, including both collected and artificial time points; (ii) we learn on more diverse data, taking into account both complete and incomplete data points; and (iii) we may balance the dataset, including incomplete data points with a particular label, into the analysis. When the dataset is imbalanced, better accuracy is expected to be obtained for a more well-represented class. However, in some applications, including classification tasks for two considered datasets, sensitivity may have greater importance than specificity. The imputation and selection of temporally distributed features are made possible by the longitudinal design of this study, in which measurements collected at multiple time points form the dataset. While gut microbiome is fairly stable over the time-span of weeks-months [68], there are intrinsic dynamics to the microbiome, specially in rapidly changing scenarios such as pregnancy and early life. Therefore, we impute the missing data by interpolating existing data from an individual and the overall distribution of data at a given time-point, with an added noise function.

We showed that feature space transformation, denoting differences in the abundances of bacteria, made by all and pivot log ratios, positively affects the accuracy of classification. These results complement the studies of [23] that used centered and isometric log ratios and concluded that there was no significant improvement in classification performance. We have demonstrated that features showing differences in bacterial abundance or in time are among the keys in prediction tasks. We have empirically shown that the Lasso regularizer [20] used in [18, 19] yields classification results that are worse than those of ANOVA for our choice of dataset.

### Application

We analyzed two datasets. For the prediction task for food allergies in infants, we obtained a balanced accuracy of 0.72, with a sensitivity of 0.66. We considered the 6th month as the last time point in the analysis. In the original work on the same dataset [11], the authors obtained a balanced accuracy of 0.66 using time points after the 6th month of infants’ life. The bacteria used for prediction are similar in our study and paper [11]. We consider the abundance collected at all time points important for the prediction. We achieved the value of accuracy via imputation of missing time points. Imputation allows the use of incomplete data with real-time points during training.

We forecast PPD via gut microbiome data collected before childbirth. We achieved a balanced accuracy of 0.62 and a sensitivity of 0.39, outperforming the accuracy of predictions on the basis of depression states observed during pregnancy and microbiome diversity. That is, we observed the predictive power of microbiome data for determining PPD. We have achieved the balanced accuracy of 0.62 using the difference in abundance of two bacteria, Veillonella parvula and Haemophilus parainfluenzae. Both species were reported to associate with mental disorders and psychotherapeutic responses [62–64]. The result is obtained by moving from compositional data to a real space via pivot log ratios [40]. All features used for classification are log ratios between species abundances. Even when a time point after childbirth is available, features important for classification are not limited by bacterial abundance measured at the last time point. We increased the value of sensitivity by imputing missing time points with only positive labels. An alternative approach to prioritize sensitivity would involve classification algorithms with guaranteed error control, as explored in [49]. The results of other articles in predicting postpartum depression [12, 28, 29] are higher than our forecasting accuracy. Notably, the last information used therein is obtained at the time of childbirth, while our feature space of interest is limited in time by the 30th week of pregnancy. A fair comparison would require microbiome data collected shortly after delivery.

Our findings complement existing results in the literature from the perspectives of the input sets and the time frame of analysis. In particular, combining microbiome data with additional background and infant-related variables could improve predictive accuracy. Also, our longitudinal analysis provides early insights on food protein-induced allergy in newborns.

While the case studies shown in this paper are both microbiome datasets, the method is applicable to any compositional dataset, including (meta-)transcriptomics and untargeted metabolomics.

### Future work

We worked with datasets where the dimensionality was greater than the size. Thus, we focused on dimensionality reduction. We merged bacteria with high sparsity and selected the optimal number of features. In such a way, we decrease the feature space to bacteria abundance or their ratios related to labels of interest. Future research could take the inverse approach by limiting the analysis to bacteria with known correlations to the labels and then applying log-ratio transformations and imputation techniques.

We used prediction accuracy as the key metric for comparing approaches. In a future work, we will focus on the imputation accuracy to to enhance the reliability of the restored values. While we assumed the same models for complete and incomplete data during imputation, further research could explore relaxing this assumption. We have applied imputation for complementing data, increasing their diversity and size, and for balancing one of the datasets. Future work should focus on oversampling techniques applicable to the considered data structure. By oversampling, that is, modeling compositional data conditional on labels, researchers can mitigate imbalances in the data, such as rare conditions or underrepresented demographics.

## Data Availability

The datasets analysed during the current study are available from NCBI under BioProject PRJEB62678 (Dataset 1) and NCBI BioProject PRJNA730851 (Dataset 2). Scripts to reproduce this research are available at the GitHub link [69].

## Appendix A Alternatives for the classification algorithms

**Table 11:** Accuracy of feature ordering via the random forest classifier for Dataset 1 with PLR transformation

order	balance	metric	acc	*p*-value	sens.	spec.	n
PCA	off	median	0.54	0.01	0.18	0.9	1,2,4,3,2
	on	mean	0.55	4e-5	0.19	0.94	1,1,18,6,5

If the balance is on, the class weights are inversely proportional to the class frequencies. Only results with *p*-value less than 0.05 are shown. *n* is the optimal number of features used

**Table 12:** Accuracy for different types of feature ordering using a support vector classifier for Dataset 1 with PLR transformation

order	balance	metric	acc	*p*-value	sens.	spec.	n
ANOVA	off	mean	0.67	6e-6	0.33	1	1,1,1,1,1
		median	0.67	6e-6	0.33	1	1,1,1,1,1
	on	mean	0.67	6e-6	0.33	1	1,1,1,1,1
		median	0.67	6e-6	0.33	1	1,1,1,1,1
Lasso	on	mean	0.56	4e-3	0.27	0.85	11,20,11,12,20
		median	0.55	5e-3	0.2	0.9	1,4,9,12,17
MI	on	median	0.52±0.02	1e-5	0.8	0.25	19,2,9,1,6

If the balance is on, the class weights are inversely proportional to the class frequencies. Only results with *p*-value less than 0.05 are shown. *n* is the optimal number of features used

**Table 13:** Accuracy for different types of feature ordering via adaptive boosting for Dataset 1 with PLR transformation

order	balance	metric	acc	*p*-value	sens.	spec.	n
hierarchical	off	median	0.54	0.01	0.2	0.89	8,20,2,12,7
ANOVA	off	mean	0.68	6e-5	0.47	0.9	5,20,13,5,1
		median	0.68	2e-7	0.4	0.95	6,1,13,2,4
PCA	off	mean	0.56	6e-4	0.27	0.86	1,3,17,16,8
MI	off	mean	0.56±0.15	0.03	0.2	0.92	6,20,3,11,13

Only results with *p*-value less than 0.05 are shown. The class weights are equal for the chosen classification method. *n* is the optimal number of features used

In the main text, we apply ANOVA to select meaningful classification features. The option of using dimensionality reduction via feature selection is justified here. We first test the (hierarchical) order of features suggested by a hierarchical tree while applying the pivot log ratio. We also consider the order given by Lasso regularization, Mutual Information (MI), and by principal component analysis (PCA) in Table 11. PCA applied to CLR provides another way to transform compositional data called the isometric log ratio [39]. Tables 12 and 13 present the results when other classifiers are applied, namely, support vector classification (SVC) and adaptive boosting (AdaBoost). The AdaBoost classifier is based on a decision tree, but then it reweighs data points depending on the correctness of the classification.

PCA, Lasso, and hierarchical ordering do not improve the accuracy. The results for the hierarchical order are as expected, as the important features are not selected in the same way as in the ANOVA method. As seen in the tables, different classification methods do not lead to an increase in balanced accuracy. From the comparison analysis, we find that adaptive boosting classifiers can improve the sensitivity of the classification when the number of features is constrained.
